# Application of the intervention Complexity Assessment Tool for Systematic Reviews within a Cochrane review: an illustrative case study

**DOI:** 10.12688/hrbopenres.13044.1

**Published:** 2020-06-01

**Authors:** Cathal A. Cadogan, Audrey Rankin, Simon Lewin, Carmel M. Hughes

**Affiliations:** 1 School of Pharmacy and Biomolecular Sciences, Royal College of Surgeons in Ireland, Dublin, Ireland; 2 School of Pharmacy, Queen’s University Belfast, Belfast, UK; 3 Division of Health Services, Norwegian Institute of Public Health, Oslo, Norway; 4 Health Systems Research Unit, South African Medical Research Council, Cape Town, South Africa

**Keywords:** intervention complexity, intervention complexity assessment tool, iCAT_SR, systematic review, evidence synthesis, complex interventions, polypharmacy, older people

## Abstract

**Background:** The intervention Complexity Assessment Tool for Systematic Reviews (iCAT_SR) has been developed to facilitate detailed assessments of intervention complexity in systematic reviews. Worked examples of the tool’s application are needed to promote its use and refinement. The aim of this case study was to apply the iCAT_SR to a subset of 20 studies included in a Cochrane review of interventions aimed at improving appropriate polypharmacy in older people.

**Methods:** Interventions were assessed independently by two authors using the six core iCAT_SR dimensions: (1) ‘Target organisational levels/categories’; (2) ‘Target behaviour/actions’; (3) ‘Active intervention components’; (4) ‘Degree of tailoring’; (5) ‘Level of skill required by intervention deliverers’; (6) ‘Level of skill required by intervention recipients’. Attempts were made to apply four optional dimensions: ‘Interaction between intervention components’; ‘Context/setting’; ‘Recipient/provider factors’; ‘Nature of causal pathway’. Inter-rater reliability was assessed using Cohen’s Kappa coefficient. Disagreements were resolved by consensus discussion. The findings are presented narratively.

**Results:** Assessments involving the core iCAT_SR dimensions showed limited consistency in intervention complexity across included studies, even when categorised according to clinical setting. Interventions were delivered across various organisational levels and categories (i.e. healthcare professionals and patients) and typically comprised multiple components. Intermediate skill levels were required by those delivering and receiving the interventions across all studies. A lack of detail in study reports precluded application of the iCAT_SR’s optional dimensions. The inter-rater reliability was substantial (Cohen's Kappa = 0.75)

**Conclusions:** This study describes the application of the iCAT_SR to studies included in a Cochrane systematic review. Future intervention studies need to ensure more detailed reporting of interventions, context and the causal pathways underlying intervention effects to allow a more holistic understanding of intervention complexity and facilitate replication in other settings. The experience gained has helped to refine the original guidance document relating to the application of iCAT_SR.

## Introduction

Several definitions of the term ‘ *complex intervention*’ exist in the literature. For example, the UK Medical Research Council has defined ‘ *complex interventions*’ as “ *interventions that contain several interacting components*” while recognising that there are multiple dimensions of complexity (e.g. the number of intervention components, interactions between components, target behaviours, target groups or organisational levels, outcomes and degree of tailoring permitted) ^1^. It is recognised that a broader understanding of the term ‘ *complex intervention*’ is required. For example, Moore *et al*. contend that “ *all interventions are complex, but some are more complex than others*” and that “ *rather than an absolute property of new components, intervention complexity can therefore be understood as a relative construct, linked to usual practice within the system, and encompassing challenges associated with disrupting and replacing often entrenched ways of working*” ^2^. Petticrew has hypothesised that there is in fact no true categorisation of interventions as ‘simple’ or ‘complex’, and that the concepts of simplicity and complexity are instead pragmatic perspectives adopted by researchers to help describe and understand the interventions being evaluated ^3^.

Interventions targeting key healthcare issues, such as multimorbidity and the use of multiple medications (i.e. polypharmacy) in older people, are often complex in that the interventions typically involve multiple components ^4, 5^. However, this broad categorisation of interventions based on their components limits our potential to systematically compare interventions and their effects. Without a more detailed exploration of intervention complexity, it is difficult to determine if varying degrees of complexity impact on intervention effectiveness. The intervention Complexity Assessment Tool for Systematic Reviews (iCAT_SR) has been developed to facilitate a more detailed and systematic assessment of intervention complexity in systematic reviews ^6^. The tool’s development process involved a series of steps which included: drafting a list of possible intervention complexity dimensions based on a review of relevant literature; development of provisional definitions for these dimensions; discussion and consensus workshops with trialists and implementation scientists to refine and agree on the tool’s core dimensions and definitions; preliminary testing of draft versions of the tool against published intervention reports; development of a finalised version of the tool and guidance document to assist with its application following further testing and refinement. Rather than providing a definitive definition of intervention complexity, the tool focuses on dimensions of complexity that can be assessed when evaluating interventions as part of a systematic review. The first version of the tool (iCAT_SR version 1.0) comprises six core dimensions and four additional dimensions that are considered to be optional, as they may not be relevant to all interventions ( Table 1). Within each dimension, there are different levels of complexity assessments. A detailed elaboration on the tool’s dimensions is available from Lewin *et al.* ^6^ and the accompanying guidance document ^7^.

**Table 1.** Overview of iCAT_SR dimensions (version 1.0).

Core dimensions	Assessment categories *
1. Organisational levels and categories targeted by the intervention	I. Multi-level II. Multi-category III. Single category
2. Behaviour or actions of intervention recipients or participants to which the intervention is directed	I. Multi-target II. Dual target III. Single target IV. Varies
3. Active components included in the intervention, in relation to the comparison	I. More than one component and delivered as a bundle II. More than one component III. One component IV. Varies
4. The degree of tailoring intended or flexibility permitted across sites or individuals in applying or implementing the intervention	I. Highly tailored/flexible II. Moderately tailored/flexible III. Inflexible IV. Varies
5. The level of skill required by those delivering the intervention in order to meet the intervention objectives	I. High level skills II. Intermediate level skills III. Basic skills IV. Varies
6. The level of skill required for the targeted behaviour when entering the included studies by those receiving the intervention, in order to meet the intervention objectives	I. High level skills II. Intermediate level skills III. Basic skills IV. Varies
Optional dimensions	Assessment categories*
7. The degree of interaction between intervention components, including the independence/ interdependence of intervention components	I. High level interaction II. Moderate interaction III. Independent IV. Varies V. Unclear/unable to assess
8. The degree to which the effects of the intervention are dependent on the context or setting in which it is implemented	I. Highly context dependent II. Moderately context dependent III. Independent of context IV. Varies V. Unclear/unable to assess
9. The degree to which the effects of the intervention are changed by recipient or provider factors	I. Highly dependent on individual-level factors II. Moderately dependent on individual-level factors III. Largely independent of individual-level factors IV. Varies V. Unclear/unable to assess
10. The nature of the causal pathway between the intervention and the outcome it is intended to effect	I. Pathway variable, long II. Pathway linear, long III. Pathway linear, short IV. Varies V. Unclear/unable to assess

*Categorisation of assessment levels: I = high; II = intermediate; III = low; IV = varies; V= unclear/unable to assess

Application of iCAT_SR in the context of systematic reviews may ultimately help researchers to consider dimensions of intervention complexity that have previously been overlooked so as to guide the pooling of studies for analysis and enhance the interpretation of review findings ^6^. Worked examples of the tool’s application are needed to promote its use and refinement. However, to date, references to the application of iCAT_SR primarily relate to protocols of ongoing systematic reviews ^8– 10^.

This case study is a proof-of-concept of the potential application of iCAT_SR to studies included in a Cochrane review. The aim was to apply the intervention Complexity Assessment Tool for Systematic Reviews (iCAT_SR) to a subset of interventions included in a Cochrane systematic review of interventions aimed at improving appropriate polypharmacy in older people ^11^. In this paper, we report on our experiences of applying iCAT_SR to included studies and outline potential refinements to the tool to facilitate its future application as part of systematic reviews.

## Methods

A convenience sample of intervention studies included in a recent update of a Cochrane review of interventions to improve appropriate polypharmacy in older people ^12^ was assessed using the iCAT_SR ^6^. The sample comprised all 20 included studies following the first round of database searches conducted as part of the review’s update. The review followed the Cochrane Collaboration’s methodology for updates of reviews. Key information relating to the review’s PICO (Population, Intervention, Comparison, Outcomes) is summarised in Table 2 and detailed information on all aspects of the methods used is available in the published review ^11^.

**Table 2.** Summary of key inclusion criteria for Cochrane review of interventions to improve appropriate polypharmacy in older people.

Study features	Key inclusion criteria
Population	Older people (≥65 years) in any healthcare setting with more than one long-term medical condition and receiving polypharmacy (≥4 medications)
Intervention	All types of interventions that aimed to improve appropriate polypharmacy in older people in any healthcare setting were eligible for inclusion provided that a validated tool was used to assess the appropriateness of prescribing
	Studies using expert opinion alone to assess the appropriateness of prescribing were excluded
Comparison	Interventions had to be compared against usual care as defined by the study (except for interrupted time series studies)
Outcomes	Primary outcomes I. Medication appropriateness (as measured by an implicit tool, such as the Medication Appropriateness Index ^35^) II. Potentially inappropriate medications (as defined by a validated explicit tool such as STOPP (Screening Tool of Older People’s Prescriptions) criteria ^36^) which could consist of the number of potentially inappropriate medications and/or the proportion of patients with one or more potentially inappropriate medications III. Potential prescribing omissions (as defined by a validated explicit tool such as START (Screening Tool to Alert to Right Treatment) criteria ^36^) which could consist of the number of potential prescribing omissions and/or the proportion of patients with one or more potential prescribing omissions IV. Hospital admissions (which included all-cause hospital admissions and unplanned hospital readmissions) Secondary outcomes V. Medication-related problems (e.g. adverse drug reactions, drug-drug interactions) VI. Adherence to medication VII. Quality of life
Study designs	Randomised controlled trials (RCTs), cluster RCTs, non-randomised trials, controlled before-and-after studies and interrupted time series

### Assessment of intervention complexity using the iCAT_SR

Assessment of intervention complexity was performed independently by two reviewers (CC, AR) using the iCAT_SR ( Table 1). Both reviewers are experienced health services researchers who led on the most recent update of the Cochrane review and, therefore, had in-depth knowledge of the included studies. In order to mitigate against a lack of detailed reporting in published study reports, study authors were emailed to request further information (e.g. intervention protocols).

For each intervention, key information was extracted using a purposefully developed data extraction form ( *Extended data* File 1 ^13^). In completing the assessments, the iCAT_SR guidance document ^7^ was used as the coding manual. For each complexity dimension, the assessment level and criteria detailed in the guidance document were applied. The coders identified relevant information from the description of the intervention under each assessment dimension, assigned a complexity rating and provided support for their assessments. Notes were taken during the coding process on any issues with applying the iCAT_SR based on the assessment levels/criteria and any refinements that were needed for the guidance document.

To aid graphical presentation of results, assessment levels across each dimension were categorised as ‘high’, ‘intermediate’, ‘low’ or ‘unclear’ using the definitions in the published tool ( Table 1). Cohen’s Kappa coefficient was used to assess inter-rater reliability between the two reviewers (≤0.2 = poor agreement, 0.21–0.40 = fair agreement, 0.51–0.6 = moderate agreement, 0.61–0.8 = substantial agreement, 0.81–1.00 = good agreement) ^14^. Any disagreements were resolved by discussion with another member of the research team (SL) who led on the development of iCAT_SR ^6^.

## Results

The characteristics of the subset of 20 studies ^15– 34^ to which the iCAT_SR was applied are summarised in *Extended data* File 2 ^13^. Briefly, these studies consisted of 12 randomised controlled trials (RCTs), six cluster RCTs and two controlled before-after studies. In total, 25,674 older patients were involved, the majority of whom were female (65.8% in intervention groups, 65.6% in control groups). On average, patients were 77.2 years old and receiving nine medicines at baseline. The studies were conducted in three types of settings: hospitals (outpatient clinics, hospital/care home interface, inpatient settings), primary care and nursing homes/residential care settings. The studies were carried out in ten countries: Australia (three studies), Belgium (two studies), Canada (two studies), Finland (one study), Germany (two studies), Ireland (two studies), Israel (one study), Italy (one study), Spain (one study) and the USA (five studies).

### Interventions

Overall, 19 studies examined pharmaceutical care-based interventions across various settings. Pharmaceutical care reflects a systematic approach to the provision of care that ensures patients receive the correct medication, at appropriate doses, for appropriate indications. It typically involves medication reviews by pharmacists in collaboration with physicians, patients and carers ^37^. One study ^29^ evaluated a single component intervention in the form of computerised decision support that was provided to general practitioners (GPs) in their own practices. Further details about the interventions are summarised in *Extended data* File 2 ^13^.

### iCAT_SR complexity assessments

Overviews of the intervention complexity assessments across the six core dimensions for the 20 studies are displayed in Table 3. A brief outline of the assessments under each of the iCAT_SR dimensions is provided in the subsections below. A detailed breakdown of individual iCAT_SR assessments for each study (including justifications for assigned ratings) is provided in *Extended data* File 3 ^13^. The inter-rater reliability was substantial (Cohen's Kappa = 0.75) ^14^.

**Table 3.** Assessments of core iCAT_SR dimensions for individual studies of interventions aimed at improving appropriate polypharmacy in older people.

Study ID grouped by setting	iCAT_SR assessment dimension					
	Organisational levels/categories	Behaviour or actions	Active components	Level of tailoring	Skill level required by those delivering intervention	Skill level required by those receiving intervention
**Community**						
Clyne 2015	High	High	High	Intermediate	Intermediate	Intermediate
Tamblyn 2003	Low	High	Low	Intermediate	Intermediate	Intermediate
Taylor 2003	High	High	High	Intermediate	Intermediate	Intermediate
**Hospital**						
Basger 2015	High	High	High	Intermediate	Intermediate	Intermediate
Bucci 2003	High	High	Intermediate	High	Intermediate	Intermediate
Crotty 2004b	Intermediate	High	High	High	Intermediate	Intermediate
Dalleur 2014	Low	High	High	Intermediate	Intermediate	Intermediate
Franchi 2016	Low	High	High	Low	Intermediate	Intermediate
Gallagher 2011	Low	High	High	Intermediate	Intermediate	Intermediate
Hanlon 1996	High	High	High	High	Intermediate	Intermediate
Michalek 2014	Low	High	High	High	Intermediate	Intermediate
Schmader 2004	High	High	High	High	Intermediate	Intermediate
Spinewine 2007	High	High	High	High	Intermediate	Intermediate
Wehling 2016	Low	High	High	High	Intermediate	Intermediate
**Residential** **care**						
Crotty 2004a	Intermediate	High	High	High	Intermediate	Intermediate
Frankenthal 2014	Low	High	High	Intermediate	Intermediate	Intermediate
Garcia- Gollarte 2014	Low	High	High	Low	Intermediate	Intermediate
Pitkala 2014	Intermediate	High	High	Low	Intermediate	Intermediate
Trygstad 2005	Low	High	High	Intermediate	Intermediate	Intermediate
Trygstad 2009	Low	High	Intermediate	Intermediate	Intermediate	Intermediate

Efforts were made to apply the iCAT_SR optional dimensions. However, a lack of detailed information in the study reports made it difficult to apply the iCAT_SR’s optional dimensions consistently across the included studies. For example, none of the included studies reported on the interaction between intervention components or the nature of the causal pathway between intervention components and outcomes.

### Organisational levels/categories

Half of the interventions targeted a single category of individuals (n=10, low level complexity rating), comprising healthcare professionals or patients. In the other studies, interventions were categorised as multi-level (n=7, high level complexity rating) when the interventions typically targeted both patients and healthcare professionals, or multi-category (n=3, intermediate level complexity rating), where multiple groups of healthcare professionals (i.e. GPs, pharmacists, nurses) within the same setting were targeted.

### Target behaviours/actions and active components

All of the interventions were deemed to be multi-target (high level complexity rating) in that they involved multiple target behaviours/actions. In all studies, this included appropriate prescribing for older patients receiving polypharmacy and the component behaviours/actions (e.g. reviewing prescriptions, implementing prescribing changes).

With the exception of one study, all of the interventions involved more than one component. Tamblyn *et al.* evaluated an intervention comprising computerised decision support which was categorised as a single component intervention (low level complexity rating) ^29^. The interventions involving more than one component were further subcategorised according to whether there was a defined order to the delivery of intervention components. There were 17 interventions delivered as a bundle (as opposed to an intervention package) as there was an order/sequence to the delivery of the interventions components (high level complexity rating). This was evident in a number of the hospital-based studies that involved the application of prescribing criteria to identify potentially inappropriate medications, which were then communicated to relevant members of a patient’s medical team and prescribing changes subsequently implemented ^19, 22^.

In two studies ^16, 32^, there was no apparent order to the delivery of intervention components and the interventions were therefore categorised as packages as opposed to intervention bundles (intermediate level complexity rating). For example, the multi-level intervention evaluated by Bucci *et al*. ^16^ targeted both patients and prescribers. Directive guidance was provided to patients with the intention of improving medication adherence, while prescribers were targeted by the pharmacist-led intervention to improve the appropriateness of medication prescribing. However, the order in which the intervention components were delivered was not explicitly stated and it was not clear if the patient-targeted component came, or needed to come, before or after the prescriber-targeted component.

### Level of tailoring

Eight interventions were categorised as highly tailored/flexible (high level complexity rating). These interventions typically involved the application of implicit tools (e.g. Medication Appropriateness Index ^35^) in assessing the appropriateness of patients’ medications. Given the judgement-based nature of these types of tools, a higher degree of tailoring/flexibility would have been permitted when being applied at the individual patient level.

Nine interventions were categorised as moderately tailored/flexible (intermediate level complexity rating). These interventions typically involved the application of explicit tools in assessing the appropriateness of patients’ medications (e.g. STOPP/START ^36^, Beers’ criteria ^38^). The rationale for rating explicit tools as moderately flexible was that while they consist of criteria relating to potentially inappropriate prescribing in older people, it is at clinicians’ discretion as to the whether the criteria are applicable to individual patients. Therefore, although these tools consist of pre-specified criteria, there is a degree of flexibility in terms of their application at the individual patient level.

The three interventions categorised as inflexible (low level complexity rating) involved education and training for healthcare professionals ^20, 23, 26^. In contrast to the studies involving prescribing tools, the intention with these interventions would be to deliver a defined and clearly specified learning activity which would have arguably less flexibility once it had been developed.

### Skill level required by those delivering and receiving interventions

For all 20 studies, assessments of the level of skill for those delivering and receiving interventions were rated as intermediate (intermediate level complexity rating). In both instances, the target behaviours/actions related to appropriate prescribing for older people. This was considered to be within the scope of normal practice of those involved in delivering and/or receiving the interventions (e.g. physicians working in geriatric hospital wards reviewing older patients’ medications), such that no specialisation was deemed to have been required.

## Discussion

This study provides the first detailed overview of the application of the iCAT_SR ^6^. The experience gained (discussed below) may assist with the tool’s use in systematic reviews in other clinical areas. The findings also demonstrate that interventions categorised as ‘multifaceted’ (comprising two or more components) in previous iterations of this review ^4, 39^ varied in complexity using the tool’s core assessment dimensions. This highlights how broad terms, such as uni-faceted and multi-faceted, do not adequately describe the scope of intervention complexity and further illustrates the importance of considering a range of dimensions of complexity using a tool such as iCAT_SR.

### Experience in applying iCAT_SR

Despite detailed available guidance on the tool’s application ^7^, this work was not without challenges. For example, time needed to be allocated to upskilling the review team on the iCAT_SR and then applying it to the interventions. Hence, this initial coding exercise focused on a subset of studies identified following the initial round of searches for the most recent update of this Cochrane review ^12^. Therefore, this work is not intended as a definitive assessment of intervention complexity in this field of research but as proof of concept of the iCAT_SR’s application.

The inter-rater reliability was substantial. Most of the observed variation between the individual rater assessments was primarily attributable to differences in the interpretation of the two skill-related dimensions (i.e. skill level required by those delivering or receiving the intervention) between the coders and whether they represented intermediate or high level skills. In assessing these dimensions, it is important to consider the baseline level of skill that would be expected of the individual(s) delivering or receiving the intervention and to note whether the study reports on the skill level required or possessed. For instance, if a study reports that individuals delivering an intervention were highly skilled and qualified/experienced, this does not necessarily mean that this level of skill/experience was required to deliver the intervention. Assessments based on the reported level of skill/experience as opposed to the required skill level could result in the complexity level assessment for this dimension being overestimated.

For the purpose of this coding exercise, appropriate prescribing for older people was deemed to be within the scope of practice of those involved in delivering and/or receiving the interventions (e.g. physicians working in geriatric hospital wards reviewing older patients’ medications), such that no specialisation was considered necessary. Consequently, all skills-related assessments were rated as intermediate. Assessments of skill level requirements should ideally be based on details of prior training. However, this may not always be explicitly outlined in study reports, in which case a judgement is required based on expected baseline skill level. In such cases, we recommend specifying *a priori* how decisions will be made between each of the assessments levels.

An additional challenge in applying the tool’s coding manual related to the assessment of the target behaviour (i.e. prescribing of appropriate polypharmacy) across included studies. The more precisely a behaviour is defined, the greater the specificity of the barriers and facilitators identified (e.g. reducing overprescribing of benzodiazepines, a commonly identified class of potentially inappropriate medications in older people); however, cases exist where it is not possible to isolate and target one behaviour for change, particularly where multiple interdependent behaviours exist (e.g. ensuring prescribing of appropriate polypharmacy for older people) ^40, 41^. Unless explicitly outlined at the outset for any given intervention, compiling an exhaustive list of these interdependent behaviours (also referred to as sub-behaviours) is practically impossible ^41^. This was the case with regard to the prescribing of appropriate polypharmacy – although these interventions typically involved medication reviews and implementation of prescribing changes, this represented an over-simplification of the key behaviours/actions. As such, the intricacies of the nursing home-based interventions that involved multi-disciplinary case conferences was not adequately captured ^18, 34^. Consequently, the target behaviour across all included studies was categorised as multi-target. However, this detracted from the potential of this core complexity dimension to discriminate between interventions. Reporting on the behaviours and actions taken as part of interventions more explicitly in future research may help in discriminating more clearly between interventions. In the interim, future research involving application of iCAT_SR may look to prioritise key expected behaviours or actions across interventions ^40^.

Finally, a lack of detailed reporting and the absence of an explicit theoretical underpinning across the interventions precluded consistent application of the iCAT_SR’s optional dimensions. These are both well recognised issues with the existing literature ^42– 47^. Consequently, assessments were largely based on the reported interventions without detailed consideration of the context/settings in which they were delivered, the interaction between intervention components and the nature of the causal pathway between intervention components and outcomes. Addressing these widely recognised issues through application of relevant reporting guidelines and checklists ^48– 51^, as well as operationalisation of appropriate theory in future research, would help to ensure more consistent application of the tool’s optional dimensions. This could enhance the tool’s capability of discriminating between interventions and offering plausible explanations for sources of heterogeneity between studies. Reporting on interactions between intervention components and the nature of the causal pathways underlying intervention effects would enhance our understanding of not just what interventions work, but also in understanding what happens when they are implemented ^52^. This could ultimately enable systematic reviews of interventions to extend beyond assessing whether interventions are effective or not, to interrogating the role of different intervention components, and exploring how, why and for whom the intervention works, and under what circumstances ^53^.

### Interpreting iCAT_SR assessments

The iCAT_SR assessments for individual studies were consistent across three dimensions which related to behaviour and skills. This was to some extent expected as the interventions focussed on improving appropriate prescribing and were delivered by clinicians involved in the care of older patients. For the remaining dimensions, there was no consistent pattern for intervention complexity assessments across included studies even when categorised according to setting. We had considered the application of a scoring system to the iCAT_SR assessments as part of this work. However, we were unable to establish a firm evidence base on which to base one.

### Refinements to iCAT_SR

The experience gained from applying the iCAT_SR has identified two potential refinements to the tool and the associated guidance document ^6^. Firstly, the assessment dimensions in the original tool have been re-ordered to enable a more logical sequence of conceptualisation and application (i.e. starting with ‘Organisational levels/categories’ and progressing to ‘Behaviour or actions’, ‘Active components’, etc.). Secondly, additional examples have been incorporated to supplement the existing ones which were largely directed towards health system and public health interventions ^7^. These may assist with the tool’s application to systematic reviews in other clinical areas. A revised version will be published on the Cochrane Collaboration’s website at a later date.

### Relationship to other work in the field

A recent systematic review of interventions to promote active transport to school in children has also applied the iCAT_SR ^54^. The authors assigned arbitrary scores to each assessment dimension to calculate a global complexity score for each study and then assessed if there was a correlation between intervention complexity and effectiveness. Considerable variation was reported in global complexity scores across included studies and no correlation was detected between intervention complexity and effectiveness. However, the review authors noted the need for more robust methods of evaluating the relationship between intervention complexity and effectiveness.

A previous overview of systematic reviews seeking to compare the effectiveness of multifaceted interventions and uni-faceted interventions in changing healthcare professionals' behaviour found no compelling evidence that the former were more effective ^55^. The current study allowed limited comparison of uni-faceted interventions and multifaceted interventions. The computerised decision support-based intervention evaluated by Tamblyn *et al.* ^29^ that was previously described in the review as uni-faceted ^4^ showed comparable assessments across a number of complexity dimensions to other interventions that were categorised as multi-faceted. This simple dichotomy of interventions according to the presence of one component or more than one component may overlook critical dimensions of complexity that impact on effectiveness. This lends support to the view that it is difficult and probably not useful to create a simple definition of a complex intervention ^6, 56^. Therefore, it may be more appropriate to define complexity in terms of intervention characteristics as opposed to the number of intervention components alone. For example, Guise *et al*. ^57^ have defined complex interventions based on key characteristics that extend beyond the intervention and components and encompass a range of other dimensions including pathway complexity, population complexity, implementation complexity and contextual complexity. It is important to recognise that complexity is not merely a characteristic of the interventions themselves but also a feature of the systems (i.e. context, setting) in which they are delivered ^58^.

### Implications for use in future reviews

The application of the iCAT_SR was a useful exercise, as it allowed dimensions of complexity to be assessed systematically across included studies. However, the value of including iCAT_SR assessments in future updates of the review is currently unclear, in relation to the additional work involved. Further work is needed to determine if application of the iCAT_SR can help to interpret whether varying levels of complexity impact on intervention effectiveness. Systematic reviews involving larger numbers of studies with data that can be pooled in meta-analyses may allow more detailed analysis of relationships between iCAT_SR assessments and intervention effect sizes using appropriate statistical techniques such as meta-regression. It is important that other systematic reviews focusing on other intervention areas apply the iCAT_SR and report on both the findings and experiences of using the tool. This will help in continuing to refine the tool and establishing methods for determining whether varying levels of complexity impact on intervention effectiveness.

## Conclusion

This study reports on the application of the iCAT_SR to a subset of studies included in a Cochrane review of interventions to improve appropriate polypharmacy in older people. The findings show that categorisation of interventions as multi-faceted or unifaceted without a more detailed assessment of other dimensions of complexity is a potential oversimplification as it not necessarily the case that interventions with fewer components are less complex across all relevant complexity dimensions. Future research needs to ensure more detailed reporting of interventions, context and the nature of the causal pathways underlying intervention effects to allow a more holistic understanding of intervention complexity. This could assist in ensuring more informative descriptions of interventions and the context in which they are delivered, and ultimately contribute to both understanding the effects of these interventions and facilitating replication in other settings.

## Data availability

### Underlying data

All data underlying the results are available as part of the article and no additional source data are required.

### Extended data

Open Science Framework: Application of the intervention Complexity Assessment Tool for Systematic Reviews (iCAT_SR) within a Cochrane review. https://doi.org/10.17605/OSF.IO/N3KW2 ^13^.

This project contains the following extended data:

– File 1: Data extraction form
– File 2: Cochrane review summary findings
– File 3: iCAT_SR assessments for individual studies

Data are available under the terms of the Creative Commons Zero "No rights reserved" data waiver (CC0 1.0 Public domain dedication).
