# Supplementary material for: Application of the intervention Complexity Assessment Tool for Systematic Reviews within a Cochrane review: an illustrative case study
Source: HRB Open Res. 2020 Jun 1;3:31. [Version 1] doi: 10.12688/hrbopenres.13044.1 (PMC7309054; doi:10.12688/hrbopenres.13044.1)
Supplement: Peer review history

# Peer review: Reviewer response for version 1

**DOI:** 10.21956/hrbopenres.14140.r27477

**Published:** 2020-07-02

**Authors:** Daniel Cury Ribeiro

**Affiliations:** 1 School of Physiotherapy, University of Otago, Dunedin, New Zealand

Thank you for inviting me to review this interesting case study. The study aimed to apply the iCAT_SR to a subset of studies that were recently included in a Cochrane Review that assessed interventions designed to improve appropriate polypharmacy in the elderly. Authors independently assessed included studies using the iCAT_SR, assessed and reported the inter-rater reliability using the Cohen’s Kappa coefficient. The findings suggest limited consistency in intervention complexity across included studies and, importantly, highlight the lack of information within included studies. In the conclusions, the authors highlight the need for more detailed information regarding the interventions tested by original studies, the context and causal pathways through which interventions may work.

 This is an interesting and well-written study. I enjoyed reading it and I have only one minor suggestion for the authors. I hope these are useful for improving the quality of the reporting.

 Table 3: suggest adding a caption below the table, for defining what “high, intermediate and low” refer to. This is implied but it is not explicit.

Is the work clearly and accurately presented and does it cite the current literature?

Yes

If applicable, is the statistical analysis and its interpretation appropriate?

Yes

Are all the source data underlying the results available to ensure full reproducibility?

Yes

Is the study design appropriate and is the work technically sound?

Yes

Are the conclusions drawn adequately supported by the results?

Yes

Are sufficient details of methods and analysis provided to allow replication by others?

Yes

Reviewer Expertise:

Process evaluation of clinical trials.

I confirm that I have read this submission and believe that I have an appropriate level of expertise to confirm that it is of an acceptable scientific standard.

# Peer review: Reviewer response for version 1

**DOI:** 10.21956/hrbopenres.14140.r27475

**Published:** 2020-06-22

**Authors:** Marie Gaarder

**Affiliations:** 1 International Initiative for Impact Evaluation, Oslo, Norway

The aim of the study was to test the Complexity Assessment Tool for Systematic Reviews (iCAT_SR) in a subset of studies included in a Cochrane review in order to promote its use and refinement. In order to draw the right conclusions and make the right interpretations of review findings, it is important to understand and document the implementation details and the context in which interventions take place. Hence testing the extent to which the tool can be implemented and whether it adds value is important.

 Interventions were assessed using the six core iCAT_SR dimensions: (1) ‘Target organisational levels/categories’; (2) ‘Target behaviour/actions’; (3) ‘Active intervention components’; (4) ‘Degree of tailoring’; (5) ‘Level of skill required by intervention deliverers’; (6) ‘Level of skill required by intervention recipients’. Attempts were made to apply four optional dimensions: ‘Interaction between intervention components’; ‘Context/setting’; ‘Recipient/provider factors’; ‘Nature of causal pathway’.

 I have three comments:

1. As I have argued elsewhere (blog: www.3ieimpact.org/blogs/misdiagnosis-and-evidence-trap-tale-inadequate-program-design), identifying the root cause or causes of a problem is maybe the most important step to designing the correct interventions to treat it. This may be obvious but is done quite infrequently, certainly in sectors outside of medicine, and I assume the iCAT_SR is meant for wider use. What this means is that you could have two identically designed (complex) interventions focusing on the same issue, in this case aimed at improving appropriate polypharmacy in older people, yet one works and the other does not because the underlying reasons for suboptimal polypharmacy were different. I do not think that the 6 SR dimensions, even if they could be applied easily, would hence be sufficient to make the right interpretations of review findings.
2. The additional four dimensions focused on interaction, context, causal pathway etc are also all key to understanding the underlying factors that drive the current situation and the effectiveness of the intervention. It is not clear whether they directly incorporate the questions of the existence of in-depth diagnoses of the problem/root cause(s) but I would strongly recommend that they should and that this should be elevated to a separate dimension. Also, the study should explain why it was decided to add the four dimensions. They seem key (from a theoretical perspective) and it is surprising that they had not been considered among the original dimensions.
3. Given the finding that there was not sufficient information on the additional dimensions to report on these, and the fact that the first 6 dimensions are necessary but not sufficient to explain differences in effectiveness, I am surprised at the section on refinements. I would venture that the authors should go further in indicating that the tool needs to incorporate the spirit behind the additional dimensions, namely understanding context, root causes, causal pathways etc.

Congratulations to the authors for a very valuable contribution.

Is the work clearly and accurately presented and does it cite the current literature?

Yes

If applicable, is the statistical analysis and its interpretation appropriate?

Not applicable

Are all the source data underlying the results available to ensure full reproducibility?

Yes

Is the study design appropriate and is the work technically sound?

Yes

Are the conclusions drawn adequately supported by the results?

Yes

Are sufficient details of methods and analysis provided to allow replication by others?

Yes

Reviewer Expertise:

International development - impact evaluations, organizational evaluations and systematic reviews.

I confirm that I have read this submission and believe that I have an appropriate level of expertise to confirm that it is of an acceptable scientific standard.

# Peer review: Reviewer response for version 1

**DOI:** 10.21956/hrbopenres.14140.r27476

**Published:** 2020-06-15

**Authors:** Livia Puljak

**Affiliations:** 1 Center for Evidence-Based Medicine and Health Care, Catholic University of Croatia, Zagreb, Croatia

I have reviewed the manuscript titled “Application of the intervention Complexity Assessment Tool for Systematic Reviews within a Cochrane review: an illustrative case study”. The aim of this case study was to apply the iCAT_SR to a subset of 20 studies included in a Cochrane review of interventions aimed at improving appropriate polypharmacy in older people.

 My comments are as follows:

- This sample of 20 studies is rather small, and homogeneous in terms of the chosen topic. But this was a case study, so that is acceptable.
- In the abstract, it is unclear what does it mean limited consistency in intervention complexity, and whether this consistency is desired or not.
- It is commendable that there are additional details posted on OSF as supplementary files.
- I am impressed that none of the assessments in Table 3 are “unclear”. I would expect poorer reporting.
- It is acknowledged that this work is not intended as a definitive assessment of intervention complexity in this field of research but as proof of concept of the iCAT_SR’s application.
- It would be interesting to report inter-rater agreement for each individual iCAT_SR dimension.

Is the work clearly and accurately presented and does it cite the current literature?

Yes

If applicable, is the statistical analysis and its interpretation appropriate?

Yes

Are all the source data underlying the results available to ensure full reproducibility?

Yes

Is the study design appropriate and is the work technically sound?

Yes

Are the conclusions drawn adequately supported by the results?

Yes

Are sufficient details of methods and analysis provided to allow replication by others?

Yes

Reviewer Expertise:

Research methodology.

I confirm that I have read this submission and believe that I have an appropriate level of expertise to confirm that it is of an acceptable scientific standard.
